# Development of highly polymorphic simple sequence repeat markers using genome-wide microsatellite variant analysis in Foxtail millet [*Setaria italica* (L.) P. Beauv.]

**DOI:** 10.1186/1471-2164-15-78

**Published:** 2014-01-28

**Authors:** Shuo Zhang, Chanjuan Tang, Qiang Zhao, Jing Li, Lifang Yang, Lufeng Qie, Xingke Fan, Lin Li, Ning Zhang, Meicheng Zhao, Xiaotong Liu, Yang Chai, Xue Zhang, Hailong Wang, Yingtao Li, Wen Li, Hui Zhi, Guanqing Jia, Xianmin Diao

**Affiliations:** 1 Institute of Crop Sciences, Chinese Academy of Agricultural Sciences, Beijing 100081, China; 2 National Center for Gene Research, Institute of Plant Physiology and Ecology, Shanghai Institutes for Biological Sciences, Chinese Academy of Sciences, Shanghai 200233, China; 3 National Center for Plant Gene Research, State Key Laboratory of Plant Genomics, Institute of Genetics and Developmental Biology, Chinese Academy of Sciences, Beijing 100101, China; 4 College of Life Science, Hebei Normal University, Shijiazhuang 050012, China; 5 College of Agronomy, Shanxi Agricultural University, Jinzhong 030801, China

**Keywords:** Microsatellite marker, SSR development, Polymorphism, *Setaria italica*

## Abstract

**Background:**

Foxtail millet (*Setaria italica* (L.) Beauv.) is an important gramineous grain-food and forage crop. It is grown worldwide for human and livestock consumption. Its small genome and diploid nature have led to foxtail millet fast becoming a novel model for investigating plant architecture, drought tolerance and C_4_ photosynthesis of grain and bioenergy crops. Therefore, cost-effective, reliable and highly polymorphic molecular markers covering the entire genome are required for diversity, mapping and functional genomics studies in this model species.

**Result:**

A total of 5,020 highly repetitive microsatellite motifs were isolated from the released genome of the genotype 'Yugu1’ by sequence scanning. Based on sequence comparison between *S. italica* and *S. viridis*, a set of 788 SSR primer pairs were designed. Of these primers, 733 produced reproducible amplicons and were polymorphic among 28 *Setaria* genotypes selected from diverse geographical locations. The number of alleles detected by these SSR markers ranged from 2 to 16, with an average polymorphism information content of 0.67. The result obtained by neighbor-joining cluster analysis of 28 *Setaria* genotypes, based on Nei’s genetic distance of the SSR data, showed that these SSR markers are highly polymorphic and effective.

**Conclusions:**

A large set of highly polymorphic SSR markers were successfully and efficiently developed based on genomic sequence comparison between different genotypes of the genus *Setaria*. The large number of new SSR markers and their placement on the physical map represent a valuable resource for studying diversity, constructing genetic maps, functional gene mapping, QTL exploration and molecular breeding in foxtail millet and its closely related species.

## Background

Foxtail millet (*Setaria italica*) is an ancient crop that is grown worldwide in arid regions, especially in East and South Asia, Africa and Europe [1-3]. According to data from the Food and Agriculture Organization, about 30 million tons (Mt) of millet grain are produced annually (http://faostat.fao.org/). In China, the current annual growing area of foxtail millet is over 2 million hectares, with a grain yield of 6 Mt [4]. As a drought-tolerant crop, foxtail millet has the potential to become more important, especially as the climate is becoming warmer and dryer [4-6].

Its small diploid genome (~515 Mb) and inbreeding nature has led to foxtail millet becoming a model for grass functional genomics, especially in investigating plant architecture, drought tolerance, crop domestication, C_4_ photosynthesis and the physiology of bioenergy crops [7-9]. The release of the genome sequence [10,11] and a haplotype map [12] have made the use of foxtail millet as a model species more attractive.

Simple sequence repeats (SSRs), also known as microsatellites, are tandem repeats of 1 to 6 nucleotides that are present in both coding and non-coding regions [13,14]. SSRs have become a marker of choice in genotyping because of their high abundance, high level of allelic variation, co-dominant inheritance and analytical simplicity. Moreover, microsatellite markers could be effectively applied in phylogenetically related species according to their conserved sequences among diverse organisms, which will greatly benefit genetic studies of related species [15]. However, despite the use of both genomic [16-19] and transcriptional [20] sequences for generating SSRs, the number of SSR markers in foxtail millet is still not adequate for efficient genetic analyses and gene mapping studies.

The level of polymorphism of SSRs is a key factor for their efficient application, and can be affected by a number of factors, including the nucleotide motif and repeat number. SSR polymorphisms are positively correlated with the number of repeat units [21]. As reported in humans [22], rice [23,24] and *Medicago truncatula* Gaertn [25], SSRs with higher numbers of repeats tend to be more polymorphic.

The availability of the completed genome sequence of foxtail millet [10,11] provides an ideal resource for genome-wide identification of SSRs *in silico* and the development of locus-specific SSR markers in this species. Taking advantage of this resource, we identified a large number of highly polymorphic SSRs by scanning for microsatellite units with relatively higher repeat numbers in the foxtail millet genome, and then assessed the efficiency of their application as developed SSR markers. The polymorphism information content (PIC) values of the SSR markers were also characterized by amplifying genotypes of a set of *Setaria* accessions from diverse geographic origins. These SSR markers could significantly stimulate genetic and genomic studies of foxtail millet and related species, further promoting it as a novel model system for genomic study.

## Results

### Identification of microsatellite motifs in the foxtail millet genome and polymorphic SSRs determination

A total of 5,020 microsatellite fragments were characterized in the released 'Yugu1’ genomic sequences (Table 1). Chromosome 9 contained the largest number of SSRs (826), followed by chromosomes 2 (612) and 1 (607). Chromosome 7 has the least number of SSRs (372). Furthermore, the 'Di’ type of SSRs constituted the majority of microsatellites detected in 'Yugu1’ (Additional file 1: Table S1). Biased distributions of the amount of each kind of SSR motifs were detected among all nine chromosomes. For example, more dinucleotide microsatellite fragments containing AT & TA, AG & GA and CT & TC units were isolated, compared with other kinds of dinucleotide repeats. Sequences containing CG & GC motifs were rarely observed according to the rigorous scanning criteria conducted in this study (Additional file 2: Figure S1).

**Table 1:** Number of polymorphic SSRs among 'Yugu1’, 'Daqingjie’ (DQJ) and 'N10’, and designed primers

**Chromosome**	**Number of SSR sequences**	**Polymorphic *vs. *** **DQJ**		**Polymorphic *vs. *** **N10**		**SSR primer design**	**Number of Polymorphic SSRs**	**Percentage of polymorphisms**
		**Number**	**%**	**Number**	**%**			
Chr.1	607	108	17.8%	215	35.4%	72	67	93.1%
Chr.2	612	121	19.8%	238	38.9%	86	85	98.8%
Chr.3	542	159	29.3%	204	37.6%	90	85	94.4%
Chr.4	396	82	20.7%	157	39.6%	65	62	95.4%
Chr.5	604	158	26.2%	268	44.4%	90	88	97.8%
Chr.6	568	158	27.8%	261	46.0%	123	110	89.4%
Chr.7	372	86	23.1%	173	46.5%	94	86	91.5%
Chr.8	493	153	31.0%	187	37.9%	35	34	97.1%
Chr.9	826	194	23.5%	350	42.4%	133	116	87.2%
Total	5020	1219	24.3%	2053	40.9%	788	733	93.0%

**Figure 1:**
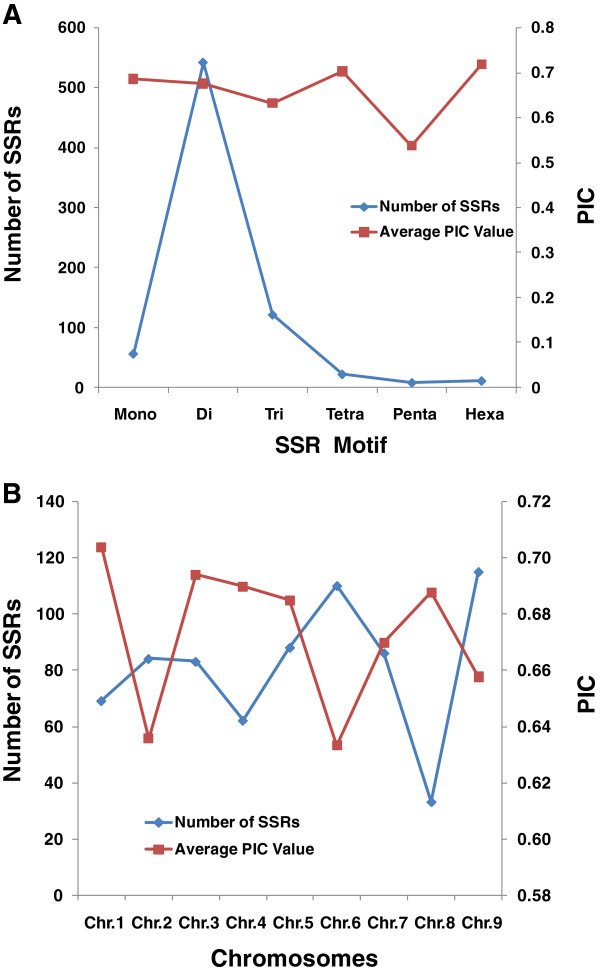
PIC variations among SSR motifs (A) and chromosomes (B).

For the efficient development of highly polymorphic SSR markers, sequence variants among 'Yugu1’, 'Daqingjie’ and 'N10’ were systematically analyzed (Table 1). The percentage of polymorphic SSRs between foxtail millet 'Yugu1’ and green foxtail 'N10’ was 40.9%, which is much higher than that between the two foxtail millet cultivars of 'Yugu1’ and 'Daqingjie’ (24.3%), indicating a higher level of polymorphism between the species (*S. italica* and *S. viridis*). In terms of diverse types of SSRs developed in this trial, a higher level of genomic variants was detected among the 'Di’ types (Figure 1A). Among the nine chromosomes of foxtail millet, the levels of SSR polymorphism and genomic variants were higher on chromosomes 9 and 6 than on the other chromosomes, although a clear difference was also observed between foxtail millet and the wild green foxtail (Figure 1B).

Based on the polymorphic microsatellite sequences identified above, 788 pairs of SSR primers were designed. Their distributions among the different chromosomes were different. The largest number was located on chromosome 9 (133), followed by chromosomes 6 (123) and 7 (94). Although there were many microsatellites in chromosome 8, only 35 pairs of SSR primers was designed because of the fewer genomic variants detected in chromosome 8 compared with the other chromosomes (Table 1).

**Figure 2:**
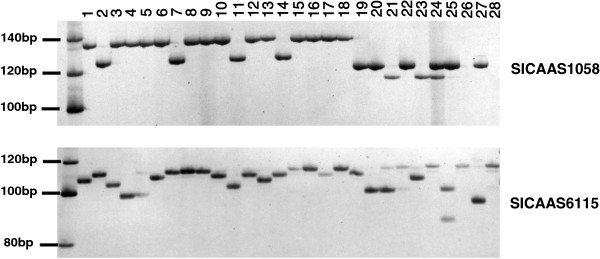
Representative electrophoresis gel showing the PCR amplification of newly developed SSR markers.

The amplification efficiency and polymorphic performance of the 788 pairs of SSR primers were assessed. The majority of the primer pairs (93.0%) produced clear and polymorphic amplicons of the expected size (Table 1; Figure 2). The number of alleles per polymorphic locus ranged from 2 to 16 (Additional file 3: Figure S2), with a median of 7. The PIC value for each locus ranged from 0.0739 to 0.9024, with a mean of 0.6687. In terms of diverse kinds of SSR motif, using the 'Di’ type of SSR as an example, GA & CT motif-containing markers gave the highest PIC value, while the CG & GC motif-containing markers showed the lowest genetic diversity among the accessions sampled in this study (Additional file 4: Table S2, Additional file 2: Figure S1).

### Validation of application efficiency and transferability of SSRs among related species of foxtail millet

**Figure 3:**
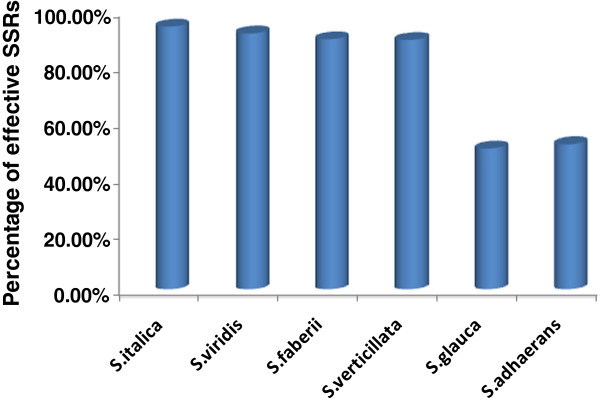
**Effectiveness of the newly developed SSRs in the *Setaria *** **genus, including *S. italica*, *S. viridis*, *S. faberii*, *S. verticillata*, *S. glauca *** **and *S. adhaerance*.**

The majority of the SSR markers (89.4%) developed from the sequence of the foxtail millet cultivar 'Yugu1’ could be effectively used in green foxtail (Figure 3), which is the wild ancestor of foxtail millet [26], implying that they share nearly identical genomes. Most of these SSRs could be used in *S. faberii* (89.6%) and *S. verticillata* (87.5%). However, only 44.7% of the SSRs could be used in *S. glauca* and *S. adhaerans*, indicating that foxtail millet is evolutionarily more distant from *S. glauca* and *S. adhaerans*.

**Figure 4:**
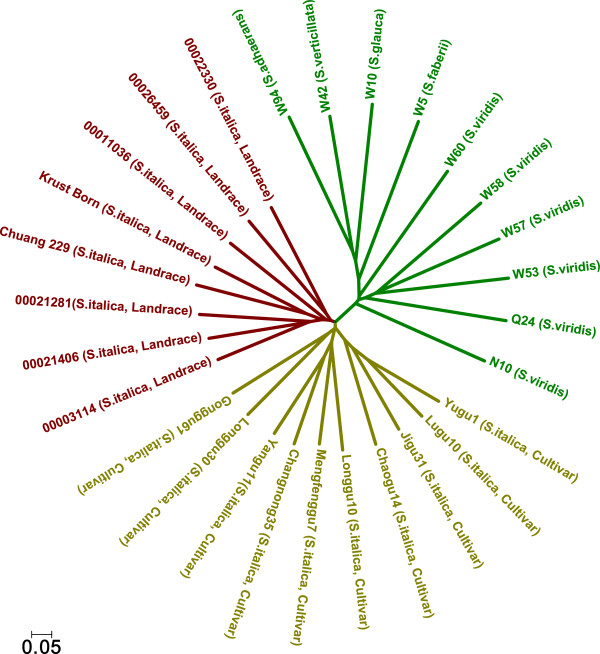
**Dendrogram of *Setaria *genus accessions inferred by 733 SSRs.** Cluster I (red), cluster II (yellow) and cluster III (green) are indicated by taxon names in different colors.

A dendrogram of the 28 *Setaria* accessions was constructed based on the polymorphic SSR data obtained in this study (Figure 4), which illustrates the phylogenetic relationships among the samples. Cluster I comprised all accessions of foxtail millet landraces. Cluster II comprised all foxtail millet cultivars sampled in this study. Cluster III comprised all other *Setaria* accessions. The genetic relationships in the dendrogram correlated well with the known *Setaria* evolutionary relationships, which indicated the value of the developed SSR markers.

### Construction of a physical map of the novel SSR markers

**Figure 5:**
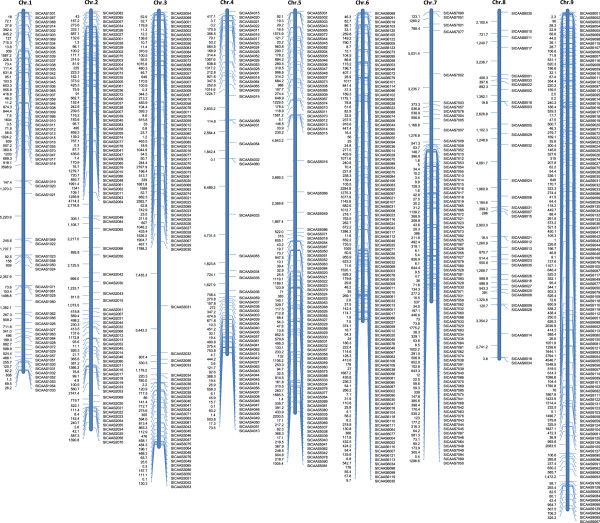
**Physical map of 733 highly polymorphic SSR markers developed in foxtail millet.** The nine chromosomes of foxtail millet are arranged from left to right, the name of each marker is shown on the right and the number on the left indicates the physical distance between neighboring markers in Kb.

A physical map of the newly developed SSR markers was constructed based on the physical distance between each pair of SSR primers (Figure 5). These markers covered the whole genome of foxtail millet, with an average distance of 550 K. Among the nine chromosomes, chromosome 6 possessed the highest density of polymorphic SSR markers (110), while chromosome 8 contained the least number of markers (34). Within each chromosome, fewer markers were found around the centromeres; most of the polymorphic markers were distally distributed on each of the chromosomes.

## Discussion

### A foxtail millet SSRs database enriched with 733 pairs of novel polymorphic SSR markers

SSRs have become a powerful marker system for genotype analysis, diversity estimation, QTL mapping and other related genetic and genomic studies [15]. However, the number of highly polymorphic SSR markers developed for foxtail millet was limited. The first set of 26 expressed sequence tag (EST)-SSRs in foxtail millet was developed in 2007 [20], which was followed by four sets of genomic SSR studies that developed 190, 45, 170, and 21,294 SSRs, respectively, using microsatellites enriched libraries [16-18] and released reference genome sequences [19]. Thus, a large set of SSRs was available for foxtail millet, which had the potential to meet the requirement of constructing high-resolution genetic maps for this model crop. However, only a small set of about 160 SSRs were evaluated for their PIC values in the *Setaria* genus in those works [16-20]. In the present study, 788 pairs SSRs were developed and all those markers were characterized based on 28 *Setaria* samples for their amplification efficiencies and polymorphism contents. Among them, 733 showed stable amplification and were highly polymorphic, with clear and available PIC values, allowing them to be anchored in the foxtail millet physical map. This large set of highly polymorphic SSR markers, combined with their corresponding physical locations, represent a valuable resource for genetic linkage map construction, QTL exploration, map based gene cloning and marker-assisted trait selection in this species. Furthermore, genome variant analysis could also be applied in studies of development of practical markers in other crop species.

### Polymorphic performance of the newly developed SSR markers

According to the polymorphism evaluation of SSRs in rice [27] and maize [28], dinucleotide repeat unit microsatellites always have larger repeat numbers and show high level of polymorphisms. Correspondingly, the dinucleotide type of SSRs developed in this study had a high average PIC value of 0.68, which was the same as that reported by Jia [16]. These values are significantly higher than those reported by others in foxtail millet [17,18,20]. This higher polymorphism performance implied that these markers could be used efficiently in foxtail millet genetic studies.

The frequency polymorphisms in GC & CG dinucleotide repeats detected in this study were low (Additional file 2: Figure S1), and similar to those reported in other crops [24,29,30]. This might be because GC-rich regions are relatively stable, resulting in less replication slippage, which generates the repeated motifs of SSRs [31], or because GC motifs are usually distributed in exons, where polymorphisms occur less frequently [29].

The majority of the highly polymorphic SSRs identified in this study were distributed in the non-coding regions of the foxtail millet genome (Additional file 5: Figure S3A, S3B). This might be a specific characteristic of highly polymorphic markers. Surprisingly, a larger proportion of the 'Tri’ type of SSRs was identified in coding regions, implying that three nucleotide insertions/deletions might be more acceptable for organisms to maintain regular growth under pressure from genomic variants occurring in coding regions. However, this hypothesis needs to be verified.

### Transferability of the developed SSRs to related species

Most of the SSR markers developed from the genome sequence of the foxtail millet cultivar 'Yugu1’ could be used in green foxtail. As the latter is the wild ancestor of domesticated foxtail millet [26], the transferability of the SSRs indicates that they share a very similar genome, although they are classified as different species botanically [5]. The phylogenetic analysis of the diverse *Setaria* accessions identified three gene pools, implying that the wild ancestor, domesticated landraces and improved cultivars of *S. italica* are distinct gene resources for breeding programs of foxtail millet. This observation is similar to that made in rice [32] and maize [33]. Previous studies of the molecular diversity of Chinese foxtail millet [34] and green foxtail [35] also support this conjecture. A large proportion of the SSRs developed in this study could also be used in *S. faberii* and *S. verticillata*, probably because these two species share the AA genome with foxtail millet. Only 44.7% of the SSRs developed in this trial could be used in *S. adhaerans* and *S. glauca*, indicating their genetic distinction from the foxtail millet AA genome. These results were consistent with those from genomic in situ hybridization analysis of the *Setaria* genomes [36]. Thus, the SSR markers developed in this study could be efficiently used in other closely related *Setaria* species.

## Conclusions

This work represents a major advance in the identification and confirmation of SSR markers for *Setaria*. A large set of 733 highly polymorphic SSR loci, with an average PIC value of 0.67, were identified by genome variants analysis based on second-generation resequencing technology.

## Methods

### Microsatellite identification

The reference genome sequence of the foxtail millet genotype 'Yugu1’ was retrieved from phytozome (http://www.phytozome.net/). SSRHunter [37] and MicroSAtellite (MISA) were used to identify microsatellite motifs (http://pgrc.ipk-gatersleben.de/misa), with the following search criteria: twenty repeat units for mononucleotide (Mono) repeats, eight (five for chromosome 6) for dinucleotide (Di) repeats, eight for trinucleotide (Tri) repeats and tetranucleotide (Tetra) repeats, and six for pentanucleotide repeats (Penta) and hexanucleotide repeats (Hexa). All selected microsatellites containing fragments were validated using the BLASTN tool in the software package ncbi-blast-2.2.25 + -win32.exe (downloaded from http://www.ncbi.nlm.nih.gov/guide/). According to the scores of all alignments for each query, a single copy was defined as the query with a top score significantly higher (at least five fold higher) than the second one. Only single copy sequences were selected for further analysis.

### Selection of polymorphic SSRs

The *S. italica* accession 'Daqingjie’ (DQJ) and the *S. viridis* accession 'N10’ were resequenced using second-generation sequencing technology with high level coverage, and the sequences obtained were *de novo* assembled [12]. The diffseq program (with default parameters) in the EMBOSS package [38] was used to compare sequence variants between the two *de novo* assemblies against the SSR sequences identified from the reference genome of 'Yugu1’. MUMmer3.22 (http://mummer.sourceforge.net/) was used to align all SSR-containing sequences with assemblies of 'DQJ’ and 'N10’, respectively, and a Perl Script was used to list the length polymorphisms. SSR containing sequences that showed polymorphisms among these genotypes were selected for primer design. Those primers that amplified a fragment between 100 bp and 300 bp were selected for further validation. Primer 3.0 (http://frodo.wi.mit.edu/) was used to design primers flanking the sequences of each unique SSR.

### Amplification efficiency and polymorphism characterization

Amplification efficiency and the level of polymorphism of the developed SSRs were assessed using 28 *Setaria* accessions originating from different parts of the world, including eight landraces of foxtail millet, ten foxtail millet cultivars*,* six accessions of green foxtail (*S. viridis*)*,* and one each of *S. glauca, S. adhaerans, S. verticillata* and *S. faberii* (Table 2). The purpose of using these closely related species was to test the transferability of the developed markers. Genomic DNA from each of the accessions was extracted using a previously described method [39]. The PCR reaction mixtures comprised 1 × Taq reaction buffer (Takara, with Mg^2+^), 125 μM each of the nucleotide dATP, dGTP, dCTP, and dTTP, 0.2 μM primers, 1 unit of Taq DNA polymerase and 50 ng of template DNA. The PCR products were initially assessed for size polymorphisms on 6% (w/w) denaturing polyacrylamide gels and visualized by silver nitrate staining. A 20 bp ladder (Takara) was used to estimate the lengths of the amplicons. The genotype data was subsequently used to determine the genetic relationships among these 28 *Setaria* accessions. PowerMarker2.5 [40] was used to construct a neighbor-joining tree based on Nei’s genetic distance (1983) [41], and the MEGA4.0 [42] was used to draw the dendrogram. The polymorphism information content (PIC) value is often used to measure the informativeness of a genetic marker [43], therefore, PowerMarker was used to evaluate the PIC value for each marker.

**Table 2:** **Sampled accessions for SSRs characterization in *Setaria***

**Trial No.**	**Accession no. or cultivar**	**Species**	**Haploid genome**^**a**^	**Origin**	**Group**
1	Krust Born	*S. italica*	A	Holland	Foxtail millet, landraces
2	Chuang229	*S. italica*	A	Missouri, US	
3	00021281	*S. italica*	A	Gansu, China	
4	00011036	*S. italica*	A	Shandong, China	
5	00021406	*S. italica*	A	Heilongjiang, China	
6	00003114	*S. italica*	A	Inner Mongolia, China	
7	00022330	*S. italica*	A	Tibet, China	
8	00026459	*S. italica*	A	Guangxi, China	
9	Yugu1	*S. italica*	A	Henan, China	Foxtail millet, cultivars
10	Jigu31	*S. italica*	A	Hebei, China	
11	Lugu10	*S. italica*	A	Shandong, China	
12	Mengfenggu7	*S. italica*	A	Inner Mongolia, China	
13	Yangu11	*S. italica*	A	Shanxi, China	
14	Longgu10	*S. italica*	A	Gansu, China	
15	Changnong35	*S. italica*	A	Shanxi, China	
16	Gonggu61	*S. italica*	A	Jilin, China	
17	Chaogu14	*S. italica*	A	Liaoning, China	
18	Longgu30	*S. italica*	A	Heilongjiang, China	
19	Q24	*S. viridis*	A	Shijiazhuang, China	Green foxtail
20	N10	*S. viridis*	A	Gansu, China	
21	W60	*S. viridis*	A	Japan	
22	W57	*S. viridis*	A	France	
23	W58	*S. viridis*	A	Oklahoma, US	
24	W53	*S. viridis*	A	Uzbekistan	
25	W5	*S. faberii*	AB	Russia	Other *setaria* species
26	W10	*S. glauca*	D	Japan	
27	W42	*S. verticillata*	AB	France	
28	W94	*S. adhaerans*	B	Spain	

a: Genome type defined in previously published works based on genomic in situ hybridization analysis [36].

### Physical map construction

BLASTN online (http://www.phytozome.net/search.php) was used to determine the physical position of each of the polymorphic SSR primers on the 'Yugu1’ genome, and the physical distances between adjacent SSRs were calculated manually. MapDraw [44] was used to construct a physical map including all the developed polymorphic SSRs.

## Abbreviations

SSR: Simple sequence repeat; QTL: Quantitative trait locus; PIC: Polymorphism information content; EST: Expressed sequence tag.

## Competing interests

The authors declare that they have no competing interests.

## Authors’ contributions

XD, GJ and MZ conceived the project and its components. JL, SZ, LY, LQ, NZ, CT, and LL designed the primers. QZ performed the polymorphic comparison. SZ, CT, XF, HW, XZ, YL, WL and HZ performed the amplification and polymorphic characterization. GJ, XD, SZ and CT analyzed the data and prepared figures and tables. GJ and XD wrote the manuscript. All authors read and approved the final manuscript.

## Supplementary Material

Additional file 1: Table S1 Number of diverse types of polymorphic (among 'Yugu1’, 'Daqingjie’ and 'N10’) SSRs developed in foxtail millet. Click here for file

Additional file 2: Figure S1 Numbers and polymorphisms of SSRs derived from diverse types of repeat units. (A) Mononucleotide SSRs; (B) Dinucleotide SSRs; (C) Trinucleotide SSRs; (D) Tetranucleotide SSRs; (E) Pentanucleotide SSRs; (F) Hexanucleotide SSRs. Click here for file

Additional file 3: Figure S2 Distribution of allele numbers for each of the polymorphic loci. The left Y-axis represents the number of markers, and the right Y-axis represents normal distributing probabilities. Click here for file

Additional file 4: Table S2 Sequences and polymorphism information for SSR primers confirmed in *Setaria* accessions. Click here for file

Additional file 5: Figure S3 Distributions of SSR motifs in coding and non-coding regions of the foxtail millet genome among motif types (A) and chromosomes (B). Click here for file
