# Supplementary material for: Development of highly polymorphic simple sequence repeat markers using genome-wide microsatellite variant analysis in Foxtail millet [Setaria italica (L.) P. Beauv.]
Source: BMC Genomics. 2014 Jan 28;15:78. doi: 10.1186/1471-2164-15-78 (PMC3930901; doi:10.1186/1471-2164-15-78)
Supplement: Additional file 1: Table S1 — Number of diverse types of polymorphic (among 'Yugu1’, 'Daqingjie’ and 'N10’) SSRs developed in foxtail millet. [file 1471-2164-15-78-S1.doc]

**Additional file 1: Table S1** Number of diverse types of polymorphic (among ‘Yugu1’, ‘Daqingjie’ and ‘N10’) SSRs developed in foxtail millet

| Type | Number of SSR sequences | Polymorphic VS DQJ | | Polymorphic VS N10 | | SSR primer design | Number of  Polymorphic SSRs | Percentage of polymorphism |
| --- | --- | --- | --- | --- | --- | --- | --- | --- |
|  |  | Number | % | Number | % |  |  |  |
| Mono | 817 | 21 | 2.5% | 44 | 5.4% | 58 | 56 | 96.6% |
| Di | 3560 | 1120 | 31.5% | 1885 | 52.9% | 566 | 515 | 91.0% |
| Tri | 525 | 59 | 11.2% | 96 | 18.3% | 122 | 121 | 99.2% |
| Tetra | 58 | 9 | 15.5% | 15 | 25.9% | 22 | 22 | 100% |
| Penta | 36 | 5 | 13.9% | 4 | 11.1% | 9 | 8 | 88.9% |
| Hexa | 24 | 5 | 20.8% | 9 | 37.5% | 11 | 11 | 100% |
| Total | 5020 | 1219 | 24.3% | 2053 | 40.9% | 788 | 733 | 93.0% |
