# Supplementary material for: Development of highly polymorphic simple sequence repeat markers using genome-wide microsatellite variant analysis in Foxtail millet [Setaria italica (L.) P. Beauv.]
Source: BMC Genomics. 2014 Jan 28;15:78. doi: 10.1186/1471-2164-15-78 (PMC3930901; doi:10.1186/1471-2164-15-78)
Supplement: Additional file 2: Figure S1 — Numbers and polymorphisms of SSRs derived from diverse types of repeat units. (A) Mononucleotide SSRs; (B) Dinucleotide SSRs; (C) Trinucleotide SSRs; (D) Tetranucleotide SSRs; (E) Pentanucleotide SSRs; (F) Hexanucleotide SSRs. [file 1471-2164-15-78-S2.doc]

**A**

**B**

**C**

**Additional file 2: Figure S1**

**D**

**E**

**F**
