# Supplementary material for: Development of highly polymorphic simple sequence repeat markers using genome-wide microsatellite variant analysis in Foxtail millet [Setaria italica (L.) P. Beauv.]
Source: BMC Genomics. 2014 Jan 28;15:78. doi: 10.1186/1471-2164-15-78 (PMC3930901; doi:10.1186/1471-2164-15-78)
Supplement: Additional file 5: Figure S3 — Distributions of SSR motifs in coding and non-coding regions of the foxtail millet genome among motif types (A) and chromosomes (B). [file 1471-2164-15-78-S5.doc]

**Additional file 5: Figure S3**

**A**

**B**
